# Neutrophil–lymphocyte ratios in the prognostication of primary non-metastatic nasopharyngeal carcinoma^☆^

**DOI:** 10.1016/j.bjorl.2017.09.004

**Published:** 2017-10-19

**Authors:** Kong Yew Liew, Abu Bakar Zulkiflee

**Affiliations:** a Universiti Putra Malaysia, Faculty of Medicine and Health Sciences, Department of Surgery, Otorhinolaryngology Unit, Serdang, Malaysia; b Universiti Malaya, Faculty of Medicine, Department of Otorhinolaryngology, Kuala Lumpur, Malaysia

**Keywords:** Nasopharyngeal carcinoma, Prognosis, Survival, Neutrophil–lymphocyte ratio, Biomarker, Carcinoma de nasofaringe, Prognóstico, Sobrevida, Relação linfócitos/neutrófilos, Biomarcador

## Abstract

**Introduction:**

Nasopharyngeal carcinoma is a geographically and racially variable disease which has a high incidence in Malaysia. Based on current concepts in tumour related inflammation the inflammatory marker, neutrophil–lymphocyte ratio was tested to find its relationship with prognosis in nasopharyngeal carcinoma.

**Objective:**

To investigate the effect of the neutrophil–lymphocyte ratio on prognosis in non-metastatic primary nasopharyngeal carcinoma patients and to further refine the cut off between high and low neutrophil–lymphocyte ratio values.

**Methods:**

The medical charts of patients with histologically confirmed nasopharyngeal carcinoma from 1st January 2005 until 31st December 2009 were reviewed retrospectively and theneutrophil–lymphocyte ratio was calculated to see if there was any association between their higher values with higher failure rates.

**Results:**

Records of 98 patients (*n* = 98) were retrieved and reviewed. Only neutrophil–lymphocyte ratio (*p* = 0.004) and tumor node metastasis staging (*p* = 0.002) were significantly different between recurrent and non-recurrent groups, with the neutrophil–lymphocyte ratio being independent of tumor node metastasis staging (*p* = 0.007). Treatment failure was significantly higher in the high neutrophil–lymphocyte ratio group (*p* = 0.001). Disease free survival was also significantly higher in this group (*p* = 0.000077).

**Conclusion:**

High neutrophil–lymphocyte ratio values are associated with higher rates of recurrence and worse disease free survival in non-metastatic nasopharyngeal carcinoma patients undergoing primary curative treatment.

## Resumo

**Introdução:**

O carcinoma de nasofaringe é uma doença variável geográfica e etnicamente, com alta incidência na Malásia. Baseado em conceitos atuais sobre inflamação relacionada a tumores, o marcador inflamatório relação neutrófilos/linfócitos foi testado para verificar sua relação com o prognóstico dessa condição clínica.

**Objetivo:**

Investigar o efeito do marcador neutrófilos/linfócitos no prognóstico de pacientes com primários não metastáticos de nasofaringe e refinar o ponto de corte entre valores altos e baixos da relação neutrófilos/linfócitos.

**Método:**

Os prontuários médicos dos pacientes com carcinoma de nasofaringe confirmado histologicamente de 1° de janeiro de 2005 até 31 de dezembro de 2009 foram revisados retrospectivamente e a relação neutrófilos/linfócitos foi calculada para verificar se havia alguma associação entre valores maiores e aumento na taxa de falha de tratamento.

**Resultados:**

Os dados de 98 pacientes (n = 98) foram revisados. Apenas a relação neutrófilos/linfócitos (*p* = 0,004) e o estadiamento TNM (*p* = 0,002) foram significantemente diferentes entre os grupos recorrentes e os não recorrentes, a relação neutrófilos/linfócitos foi independente do estadiamento TNM (*p* = 0,007). A falha de tratamento foi significantemente maior no grupo com relação neutrófilos/linfócitos alta (*p* = 0,001). A sobrevida livre de doença também foi significantemente maior nesse grupo (*p* = 0,000077).

**Conclusão:**

Os altos valores da relação neutrófilos/linfócitos estão associados a maiores taxas de recorrência e menor tempo de sobrevida livre de doença em pacientes com carcinomas não metastáticos de nasofaringe submetidos a tratamento curativo primário.

## Introduction

Nasopharyngeal Carcinoma (NPC) is a very geographically and racially variable disease. Based on World Health Organization (WHO) statistics, there are about 800,000 new cases of NPC reported yearly which account for about 0.7% of all malignancies worldwide.

## Epidemiology of Nasopharyngeal Carcinoma

The incidence of the disease in western countries is very low and in North America and Europe is less than 1 per 100,000 population.^1^, ^2^ Outside of America and Europe however the disease is endemic to parts of Southern China and South East Asian. In China the incidence is variable from northern to southern China, with low incidence in areas like Beijing and Tianjin compared to more southern locations such as in Guangdong and Hong Kong.^2^ In comparison other oriental countries, like Japan and Korea, exhibit a very low rate of NPC.^2^ In Malaysia NPC is the third most common cancer in men.^3^ The Age Standardized incidence Rate (ASR) overall for Malaysians according to the National Cancer Registry of 2006 is 8.5 per 100,000 for males and 2.6 per 100,000 for females.^3^

NPC is classified into three histological subtypes by the WHO classification: Type 1 (I) Squamous cell carcinoma, and Type 2 undifferentiated carcinoma; which is further divided into 2a (II) keratinizing undifferentiated carcinoma and 2b (III) nonkeratinising undifferentiated carcinoma.^1^ The majority, up to 95% of cases, of NPC are of WHO Type III nonkeratinising undifferentiated carcinoma which is related to high incidence areas while the remainder of the keratinizing subtypes tend to occur in low incidence areas and are considered to be of a different aetiology.^1^, ^2^

The WHO currently recommends Radiotherapy (RT) for Stage I NPC and Concurrent Chemo-Radiotherapy (CCRT) for non-metastatic Stage II–IV NPC, with or without adjuvant chemotherapy. The standard for RT is a minimum 3 Dimensional (3D) RT, with Intensity Modulated RT (IMRT) in centre's where it is available.

### General treatment outcomes in nasopharyngeal carcinoma

According to the American Joint Committee on Cancer (AJCC) 7th edition manual, 5 year survival rates for NPC by stage are: 72% for Stage I, 64% for Stage II, 62% for Stage III and 38% for Stage IV.^4^

As technology in RT has improved, survival rates have improved and toxicity rates have reduced. The conversion from 2 Dimensional (2D) RT to 3DRT and then IMRT has improved Disease Free Survival and overall survival for all stages of NPC. The RT outcomes for NPC patients between 2004 and 2008 was published by Chee Ee Phua et al. and shows Stage I 5 year survival at 81.8%, followed by 77.9%, 47.4% and 25.9% for Stage II to Stage IV respectively.^5^ Overall recurrence for all stages was 53.4%.^5^

## Cancer related inflammation

Since the 19th century there has been a link between persistent inflammation and the development of malignancy.^6^ This is evident in cases of malignancy secondary to conditions such as ulcerative colitis and in Marjolin's ulcers. It is estimated that about 25% of cancers are related to chronic inflammation.^7^ Even tumours that are not secondary to inflammation exhibit inflammatory cells within their microenvironment.^6^, ^7^

The current concept of cancer related inflammation is that there are two pathways: an intrinsic pathway, where oncogenes and tumour suppressor genes activate the expression of inflammation mediated programmers; and an extrinsic pathway where chronic inflammation leads to carcinogenesis.^6^, ^7^, ^8^ With regard to inflammation in NPC some work has been done that shows in vitro that COX-2 is involved in the multistep process of NPC carcinogenesis and the Cyclo-Oxygenase 2 inhibitor Celecoxib may play a role in inhibition of invasion and migration of NPC cells.^9^ The usage of Celecoxib was also able to enhance the effect of radiotherapy.^10^ Since inflammation is related to carcinogenesis, proliferation and invasion, are we able to identify patients within the disease population that have worse inflammation, and consequently possibly worse outcomes?

## Methods

The study was a retrospective cohort study conducted at a tertiary referral hospital in the capital of Malaysia. The study objectives were as follows:

- To establish if an elevated Neutrophil–Lymphocyte Ratio (NLR) is associated with increased treatment failure in non-metastatic Nasopharyngeal Carcinoma.
- To determine if an elevated Neutrophil–Lymphocyte Ratio (NLR) is associated with shortened time to recurrence.
- To calculate a prognostically significant value to identify high NLR, and thus poorer prognosis.

The null Hypothesis (H_0_) was that patients with elevated NLR have no greater risk for treatment failure or recurrence.

### Ethics approval

Ethics approval was obtained via the medical ethics committee of our institution (MECID no. 20148-478) prior to commencement of the study.

### Data collection

Patients who were diagnosed with Nasopharyngeal Carcinoma (NPC) from the 1st of January 2005 until the 31st of December 2009 were identified. A review of their medical records was performed looking for information on the demographic data (age, gender), staging, WHO type, neutrophil and lymphocyte plasma levels and treatment outcomes.

### Inclusion criteria

Inclusion criteria for the study were if the patients had:

- Histologically confirmed diagnosis of NPC from 1st of January 2005 until 31st of December 2009;
- Availability of pretreatment Full Blood Count (FBC) with differential counts;
- Completion of prescribed chemotherapy, radiotherapy or concurrent treatment regimes.

### Exclusion criteria

The exclusion criteria for the patients were if they had:

- Metastatic disease at the time of diagnosis;
- Presence of other concurrent primary malignancy;
- Known underlying autoimmune or inflammatory disorders;
- Active ongoing infection at the time of diagnosis;
- Incomplete NPC treatment.

### Data collected

The following data was collection from patients which met the inclusion and exclusion criteria:

- Age at diagnosis;
- Gender;
- Ethnicity;
- WHO classification of tumour;
- Staging;
- Time of diagnosis;
- Total time to recurrence, in months;
- Total follow up duration, in months;
- Percentage and absolute counts of neutrophils in the pretreatment differential count;
- Percentage and absolute counts of lymphocytes in the pretreatment differential count.

The Neutrophil–Lymphocyte Ratio (NLR) was then calculated by dividing the percentage neutrophils by the percentage lymphocytes.

### Data analysis

The data analysis was performed with SPSS version 22. Descriptive statistics of the study population was established.

The independent *t*-test was performed to establish if failure of treatment was related to NLR levels and the One-way ANOVA test was performed to determine if NLR was related to the stage of disease.

Subsequently the cutoff point for high and low NLR levels was determined from a Receiver Operating Characteristic (ROC) curve and the study population was divided into high and low NLR groups. These two groups were then further subjected to the Independent T test to identify any other significant factors that may differ between groups.

Finally Kaplan–Meier survival curves were plotted and the log rank test was done to establish survival between high and low NLR groups.

## Results

### Descriptive statistics and population demographics

A total of 98 (*n* = 98) patients were identified who fit the inclusion and exclusion criteria. Malaysia being an ethnically diverse country, the patients were divided by race initially. The majority of the patients were Chinese (81.6%), followed by Malay (11.2%), Indian (4.1%), Kadazan (1.0%), Iban (1.0%) and Eurasian (1.0%). 68.4% were male and 31.6% were female.

The mean age of the patients was 49.81 ± 12.21 years (range 21–77 years).

Of the diagnosed NPC, the majority were classified as WHO 3 tumours (62.2%) and the remainder were WHO 2 (37.8%). There were no patients with WHO 1 tumours identified in this study.

At diagnosis tumours were staged as follows: Stage I (3.1%), Stage II (28.6%), Stage III (36.7%), Stage IVa (17.3%) and Stage IVb (14.3%). 33.7% of patients failed primary treatment, either having residual locoregional disease or developed recurrence 6 months after completion of treatment. 66.3% of patients remained tumour free at the time of analysis. The mean time to recurrence was 31.18 ± 28.84 months and the mean follow up time for all patients combined was 64.85 ± 32.28 months.

The NLR for all patients ranged between 1.010 and 6.000. The mean was 2.868 ± 1.141.

### Establishing a link between NLR and failure of treatment

The study population was divided into 2 groups: (1) Those that had failed treatment (indicated by residual or recurrent disease); and (2) Those that remained tumour free.

From the failure of treatment group, the mean NLR was 3.326 ± 0.992 while the tumour free group had a mean NLR of 2.635 ± 1.147. Table 1 summarizes the factors measured between treatment failure and non-treatment failure groups.

**Table 1:** Variables between recurrences free patients and those with recurrence.

	Treatment failure (*n* = 33)	No treatment failure (*n* = 65)	*p*-value
*NLR*	3.326 ± 0.992	2.635 ± 1.147	0.004

*Race (%)*			0.514
Chinese	78.8	83.1	
Malay	12.1	10.8	
Indian	6.1	3.1	
Kadazan	0	1.5	
Iban	0	1.5	
Eurasian	3.0	0	

*Age (years)*	49.48 ± 10.072	49.97 ± 13.235	0.854

*Gender (%)*			0.114
Male	78.8	63.1	
Female	21.2	36.9	

*WHO classification (%)*			0.497
WHO 2	42.4	35.4	
WHO 3	57.6	64.6	

*Staging (%)*			0.002
I	0	4.6	
II	12.1	36.9	
III	30.3	40.0	
IVa	30.3	10.8	
IVb	27.3	7.7	

*NLR*, Neutrophil–Lymphocyte Ratio; *WHO*, World Health Organization.

The independent *t*-test was performed for treatment failure against NLR and was found to be statistically significant (*p* = 0.004), thus there is a statistically significant difference between the levels of the NLR in the treatment success and the treatment failure groups.

### Proving that the NLR is not dependent on staging

Table 2 shows the mean NLR by stage of diagnosed NPC.

**Table 2:** The mean NLR values by TNM staging.

Stage	Mean NLR	Standard deviation
I	2.257	0.645
II	2.518	1.163
III	2.706	1.032
IVa	3.183	1.064
IVb	3.733	1.102

*NLR*, Neutrophil–Lymphocyte Ratio; *TNM*, Tumor Node Metastasis staging.

Based on the above table the mean NLR is shown to increase with advancing stage. The One-way ANOVA test was then performed to test the dependence of the NLR on staging.

The result was statistically significant (*p* = 0.007), which showed that NLR and staging are not dependent factors.

### Defining the cutoff value for ‘high’ and ‘low’ NLR values

**Figure 1:**
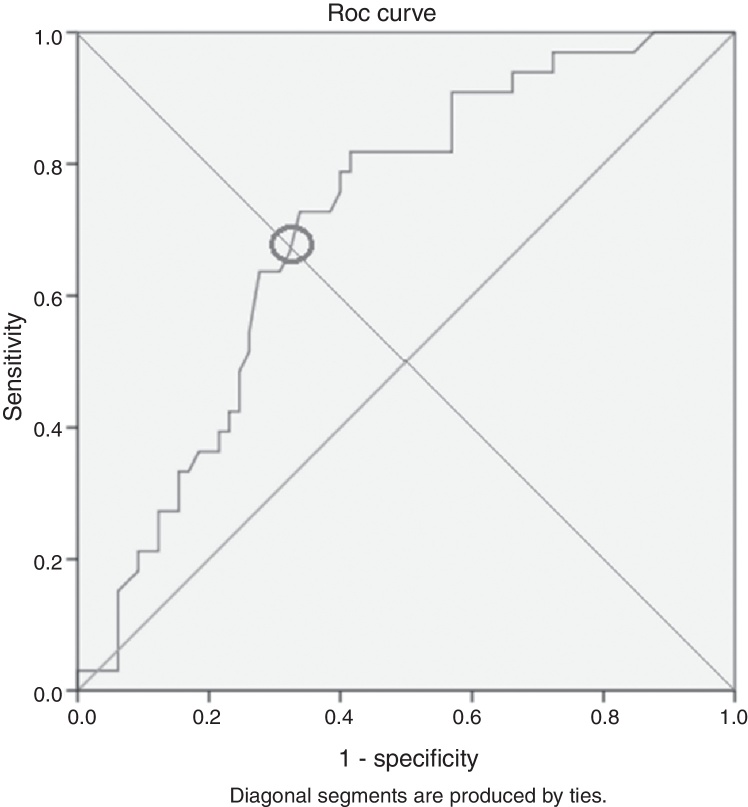
The receiver operating characteristic curve of NLR between recurrence and recurrence free groups.

A Receiver Operating Characteristic (ROC) curve was plotted and the suitable cutoff was found to be a NLR of 2.995 (*p* = 0.001) (Fig. 1).

Thus a NLR value of less than 2.995 was designated as low while values greater than 2.995 were grouped as high.

### Summary of high and low NLR groups

Table 3 summarizes the variables between the high (*n* = 43) and low (*n* = 55) NLR groups, compared against the total study population.

**Table 3:** Variables between high and low NLR groups.

	Total population	High NLR (*n* = 43)	Low NLR (*n* = 55)	*p*-value
*Race (%)*				0.070
Chinese	81.6	74.4	87.3	
Malay	11.2	14.0	9.1	
Indian	4.1	7.0	1.8	
Iban	1.0	2.3	0	
Kadazan	1.0	0	1.8	
Eurasian	1.0	2.3	0	

*Age (years)*	49.81 ± 12.21	49.19 ± 11.41	50.29 ± 12.88	0.659

*Gender (%)*				0.259
Male	68.4	74.4	63.6	
Female	31.6	25.6	36.4	

*WHO classification (%)*				0.116
WHO 2	37.8	46.5	30.9	
WHO 3	62.2	53.5	69.1	

*Staging (%)*				0.000012
I	3.1	2.3	3.6	
II	28.6	14.0	40.0	
III	36.7	30.2	41.8	
IVa	17.3	25.6	10.9	
IVb	14.3	27.9	3.6	

*Treatment failure (%)*				0.001
Yes	33.7	51.2	20.0	
No	66.3	48.8	80.0	

*Time to recurrence (months)*	31.18 ± 28.84	28.90 ± 27.12	41.00 ± 30.77	0.170
*Follow up time (months)*	64.85 ± 32.28	55.19 ± 34.05	72.40 ± 28.93	0.008

*NLR*, Neutrophil–Lymphocyte Ratio; *WHO*, World Health Organization.

### Determining the relationship between failure and high NLR

The Pearson's Chi Square test was then performed to test failure of treatment against high and low NLR groups. The result was statistically significant (*p* = 0.001) with an odds ratio of 4.190.

Thus patients with a NLR of more than 2.955 are statistically 4.19 times more likely to fail primary curative chemo or radiotherapy for Nasopharyngeal Carcinoma.

### Kaplan–Meier survival curves and Cox regression analysis

**Figure 2:**
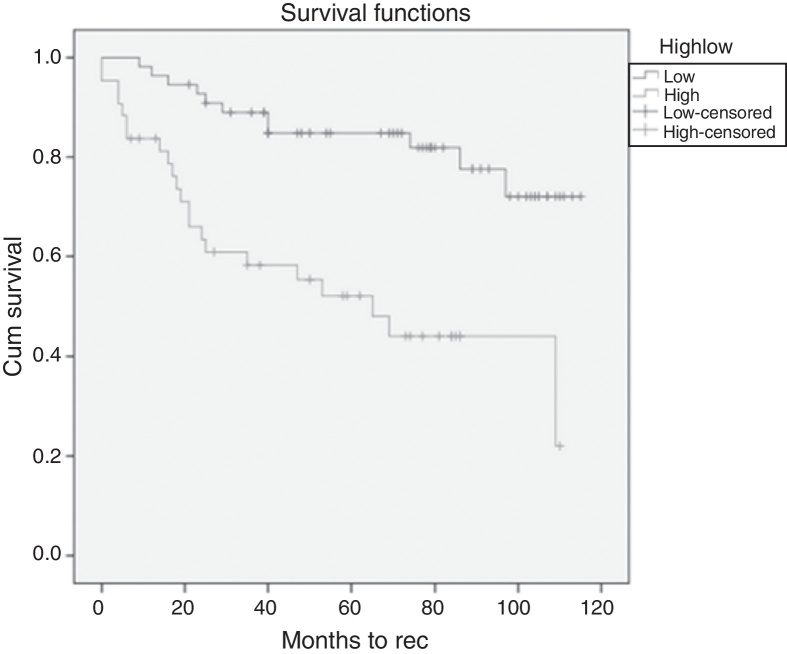
Kaplan–Meier survival curves between high and low NLR groups.

Finally the Kaplan–Meier survival curves were plotted to determine if there was any difference between the Disease Free Survival (DFS) of patients from either the high or low NLR groups. Below is the Kaplan–Meier curve for survival times between high and low NLR groups (Fig. 2).

The overall mean DFS time in months was 83.218 ± 4.527. The low NLR group had a mean DFS of 97.85 ± 4.677 months while the high NLR group had a mean DFS of 62.657 ± 7.212 months. The result was statistically significant with *p* = 0.000077.

Cox regression analysis was then performed. The Hazard Ratio (HR) 3.953 with a 95% Confidence Interval (95% CI) of 1.896–8.240. This was statistically significant with *p* = 0.000246.

## Discussion

### Significance of the Neutrophil–Lymphocyte Ratio

Since the 19th century there have been observations between inflammation and carcinogenesis. More recent work has gone to show that the acute inflammatory response plays some role in carcinogenesis as well as local and distant spread of cancers. However it was not until Walsh in 2005 demonstrated that patients with a raised Neutrophil–Lymphocyte Ratio (NLR) was associated with poorer overall and cancer specific survival that it was looked into with more detail with regard to specific cancers.^11^

Since then there have been multiple papers detailing how the NLR is useful as a prognostic indicator in various types of cancers. The majority of the work done revolved around colorectal,^11^, ^12^, ^13^ breast,^14^, ^15^ liver^16^ and renal cancers^17^, ^18^ but of late some papers have been published on NLR with regard to both Nasopharyngeal Carcinoma (NPC)^19^, ^20^, ^21^ and Head and Neck Cancers (HNC) in general.^22^, ^23^, ^24^

Almost all studies on NLR in cancer has come back with the conclusion that the higher the NLR the worse the prognosis.^11^, ^12^, ^13^, ^14^, ^15^, ^16^, ^17^, ^18^, ^19^, ^20^, ^21^, ^22^, ^23^, ^24^ This has been found to be true in both metastatic and non-metastatic cancers. However the heterogenicity of the data and the other measured variables (such as age, staging, tumour types, site) makes it difficult to recommend more than just that the NLR is a useful indicator. Important observations to note from various studies was that neutrophil and lymphocyte counts tended to be inversely proportional in nature, reflecting the balance between inflammation and angiogenesis (high neutrophils) and a protective host immune response (high lymphocytes). Several studies have noted that higher levels of neutrophils alone correlated with a worse Overall Survival (OS) while a higher lymphocyte count alone correlated with a better Progression Free Survival (PFS) and OS.

Due to the heterogenicity of the cancers and treatment modalities, we feel that NPC would be an ideal disease for testing this hypothesis against as it is a single primary site disease with a single type of primary treatment, which are radiotherapy or combined chemo and radiotherapy.

From our data we initially aimed to prove that the NLR was significantly higher in cases of failed treatment, defined as patients with either residual or recurrent disease, than in cases where the treatment was successful and patients had remained disease free. The mean NLR in the treatment failure group was higher at 3.326 ± 0.992 compared to the treatment success group which was 2.635 ± 1.147. The difference between the mean NLR of both groups was statistically significant on the independent *t*-test (*p* = 0.004) proving that the patients with failure of treatment had higher NLR.

Other factors measured between treatment success and treatment failure group in this study were age, race, staging, WHO type and gender. Only staging proved to be statistically significantly different between the treatment success and treatment failure groups (*p* = 0.002) while age (*p* = 0.854), race (*p* = 0.514), WHO type (*p* = 0.497) and gender (*p* = 0.114) were not significantly different between the two groups. As expected staging would differ between the two groups but surprisingly no other factors were found to be significantly different between the treatment failure and treatment success groups.

Some studies in NLR for non-HNC have found that the NLR is dependent on higher staging.^13^, ^15^, ^25^ A result as this would obliviate the need for NLR as Tumor Node Metastasis (TNM) staging would be equally accurate, and is already in use. However the studies done for HNC and specifically NPC found that the NLR was not dependent on the stage or other demographic data.^19^ Rassouli et al. had combined the NLR with the Platelet Lymphocyte Ratio (PLR) to come to a scoring criteria which they feel is at least as good as the TNM staging for HNC^24^ while Chang et al. suggested a scoring system based on age, gender, T and N Stages, haemoglobin levels during radiotherapy, pretreatment NLR, and platelet levels during radiotherapy which they feel is superior to the TNM staging for NPC.^26^

Our objective at this point was to prove that the NLR was independent of the TNM stage and as such would be able to be used as an independent prognostic indicator for NPC. The descriptive data does generally show that the mean NLR increased with the corresponding increase in TNM stage, so the One-way ANOVA was run to determine exactly if the NLR was dependent on this. The *p* value for the One-way ANOVA was 0.007 which shows that statistically the NLR is independent of the staging.

The original study by Walsh et al. quoted a high NLR as being more than 5 while a low NLR as being less than 5.^11^ Further studies have either used the NLR value of 5 or from calculations have come up with a different value. This value ranges from 2.2 to 4.27. Specifically for NPC, An et al. had calculated their cutoff value as 3.73.^19^ Other papers have used the NLR as a continuous variable and expressed the value in tertiles or quartiles to give several groups, each with a slightly worse prognosis.^14^, ^20^, ^21^

We had decided to try to establish a cutoff value to define ‘high’ and ‘low’ NLR in order to allow easier prognostication during clinical work as no ‘true’ normal values have been given for the NLR prior to this. From the ROC curve the value found was 2.955, which was significant with a *p* = 0.001. This NLR value is fairly close to several other studies such as a meta-analysis for Renal Cell Carcinoma (RCC) by Hu et al.,^17^ and a breast cancer study by Krenn-Pilko et al.,^15^ which found the cutoff NLR to be 3.

When the study population is divided into the high and low NLR groups, it was found that age, gender, race, WHO type, time to recurrence and follow up durations were not significantly different between the two groups. There was significant differences between the staging (*p* = 0.000012) and treatment failure (*p* = 0.001) between the two groups, indicating that patients with high NLR are significantly more likely to have higher stage disease and significantly more likely to have treatment failure.

The Oodds Ratio (OR) for failure in the high NLR group was 4.190, thus meaning that patients with a NLR value of more than 2.955 are 4.19 times more likely to have residual or recurrence disease after completion of curative radiotherapy or concurrent chemoradiotherapy.

Other measured factors in this study were age, race, staging, WHO type and gender. Only staging proved to be statistically significantly different between the treatment success and treatment failure groups (*p* = 0.002) while age (*p* = 0.854), race (*p* = 0.514), WHO type (*p* = 0.497) and gender (*p* = 0.114) were not significantly different between the two groups. As expected staging would differ between the two groups but surprisingly no other factors were found to be significantly different between the treatment failure and treatment success groups.

### Comparing the NLR to other methods of prognostication

In recent years a lot of effort has gone into genomics and molecular testing to assist with prognostication of different types of cancers, including NPC. One of the biomarkers that have received the most attention recently is Epstein–Barr Virus (EBV) DNA testing. This is done via Polymerase Chain Reaction (PCR) and there is some evidence to suggest that it is a useful prognosticator for NPC treatment. Studies have found that the EBV DNA titres are likely to correspond to tumour load^27^ and have been shown to be an independent prognosticator for recurrence and distant metastases, with levels rising up to six months before clinical detection of recurrence.^28^ High mid treatment levels have also been found to be associated with treatment failure.^29^ Unfortunately the EBV DNA was found to be detectable in 93% of tumours, and only in non-keratinising (WHO 2 and 3) types.^29^ It is a useful prognosticator for tumours where the titres are detectable, but not suitable in all cases. Also the availability of PCR and the cost of testing for EBV DNA has yet to be evaluated and may be an issue within the Malaysian healthcare system.

High sensitivity C-Reactive Protein (hs-CRP) has also been studied as a biomarker to complement EBV DNA.^30^ Hs-CRP has been used mainly in cardiac studies for prognostication of patients with ischaemic heart disease. High levels have found to give a worse prognosis for gastroesophageal cancers, colorectal cancers, breast cancers and inoperable lung cancers. Tang et al. found hs-CRP to be an independent marker for survival in NPC and recommended hs-CRP to be used in conjunction with EBV DNA to allow better prognostication but no scoring system was given.^30^ Unfortunately as hs-CRP is generally used as a cardiac prognosticator, there were concerns that the results were confounded by the increased cardiac risk profile of the test population. If both the hs-CRP and the EBV DNA were high then there was a quoted 5-fold increase in risk.^30^

Alone the NLR has demonstrated itself to be an independent prognosticator for NPC, but it may likely be enhanced if it was able to be incorporated into a scoring model with other readily available tests. Chang et al. has recommended a 19 point scoring system based on gender, age, T or N Stages, anaemia or thrombocytosis during radiotherapy, continuous reduction in haemoglobin and high NLR before radiotherapy but have not included their scoring system for analysis.^26^ A simplified system that may be of use involves screening using the PLR before implementing the NLR, as tested by Rassouli for HNC.^24^

## Conclusion

Given the data we conclude that a raised pretreatment NLR is a good negative prognostic indicator in NPC and accept H_1_. Patients with a NLR value of more than 2.995 have a reduced DFS with a HR of 3.953 (95% CI 1.896–8.240).

## Conflicts of interest

The authors declare no conflicts of interest.

## References

[1] Chan J.K.C., Bray F., McCarron P., Foo W., Lee A.W.M., Yip T., Barnes L., Evenson J.W., Reichart P., Sidransky D. (2005). Pathology and genetics of head and neck tumours.

[2] Chang E., Adami H. (2006). The enigmatic epidemiology of nasopharyngeal carcinoma. Cancer Epidemiol Biomarkers Prev.

[3] Aminuddin M.Y. (2011). Health technology assessment report: nasopharyngeal carcinoma screening.

[4] American Joint Committee on Cancer, Edge S.B., Byrd D.R., Compton C.C., Fritz A.G., Green F.L., Trotti A. (2010). AJCC cancer staging manual.

[5] Chee Ee Phua V., Loo W.H., Yusof M.M., Wan Ishak W.Z., Tho L.M., Ung N.M. (2013). Treatment outcome for nasopharyngeal carcinoma in University Malaya Medical Centre from 2004–2008. Asian Pac J Cancer Prev.

[6] Mantovani A., Allavena P., Sica A., Balkwill F. (2008). Cancer-related inflammation. Nature.

[7] Balkwill F.R., Mantovani A. (2012). Cancer-related inflammation: common themes and therapeutic opportunities. Semin Cancer Biol.

[8] Solinas G., Marchesi F., Garlanda C., Mantovani A., Allavena P. (2010). Inflammation-mediated promotion of invasion and metastasis. Cancer Metastasis Rev.

[9] Tan K.B., Putti T.C. (2005). Cyclooxygenase 2 expression in nasopharyngeal carcinoma: immunohistochemical findings and potential implications. J Clin Pathol.

[10] Zhang S.X., Qiu Q.H., Chen W.B., Liang C.H., Huang B. (2014). Celecoxib enhances radiosensitivity via induction of G_2_–M phase arrest and apoptosis in nasopharyngeal carcinoma. Cell Physiol Biochem.

[11] Walsh S.R., Cook E.J., Goulder F., Justin T.A., Keeling N.J. (2005). Neutrophil–lymphocyte ratio as a prognostic factor in colorectal cancer. J Surg Oncol.

[12] Ozdemir Y., Akin M.L., Sucullu I., Balta A.Z., Yucel E. (2014). Pretreatment neutrophil/lymphocyte ratio as a prognostic aid in colorectal cancer. Asian Pac J Cancer Prev.

[13] Galizia G., Lieto E., Zamboli A., De Vita F., Castellano P., Romano C. (2015). Neutrophil to lymphocyte ratio is a strong predictor of tumor recurrence in early colon cancers: a propensity score-matched analysis. Surgery.

[14] Koh C.H., Bhoo-Pathy N., Ng K.L., Jabir R.S., Tan G.H., See M.H. (2015). Utility of pre-treatment neutrophil–lymphocyte ratio and platelet–lymphocyte ratio as prognostic factors in breast cancer. Br J Cancer.

[15] Krenn-Pilko S., Langsenlehner U., Stojakovic T., Pichler M., Gerger A., Kapp K.S. (2016). The elevated preoperative derived neutrophil-to-lymphocyte ratio predicts poor clinical outcome in breast cancer patients. Tumor Biol.

[16] Xue T.C., Zhang L., Xie X.Y., Ge N.L., Li L.X., Zhang B.H. (2014). Prognostic significance of the neutrophil-to-lymphocyte ratio in primary liver cancer: a meta-analysis. PLOS ONE.

[17] Hu K., Lou L., Ye J., Zhang S. (2015). Prognostic role of the neutrophil–lymphocyte ratio in renal cell carcinoma: a meta-analysis. BMJ Open.

[18] de Martino M., Pantuck A.J., Hofbauer S., Waldert M., Shariat S.F., Belldegrun A.S. (2013). Prognostic impact of preoperative neutrophil-to-lymphocyte ratio in localized nonclear cell renal cell carcinoma. J Urol.

[19] An X., Ding P.R., Wang F.H., Jiang W.Q., Li Y.H. (2010). Elevated neutrophil to lymphocyte ratio predicts poor prognosis in nasopharyngeal carcinoma. Tumor Biol.

[20] He J.R., Shen G.P., Ren Z.F., Qin H., Cui C., Zhang Y. (2012). Pretreatment levels of peripheral neutrophils and lymphocytes as independent prognostic factors in patients with nasopharyngeal carcinoma. Head Neck.

[21] Jin Y., Ye X., He C., Zhang B., Zhang Y. (2015). Pretreatment neutrophil-to-lymphocyte ratio as predictor of survival for patients with metastatic nasopharyngeal carcinoma. Head Neck.

[22] Haddad C.R., Guo L., Clarke S., Guminski A., Back M., Eade T. (2015). Neutrophil-to-lymphocyte ratio in head and neck cancer. J Med Imaging Radiat Oncol.

[23] Rachidi S., Wallace K., Wrangle J.M., Day T.A., Alberg A.J., Li Z. (2016). Neutrophil-to-lymphocyte ratio and overall survival in all sites of head and neck squamous cell carcinoma. Head Neck.

[24] Rassouli A., Saliba J., Castano R., Hier M., Zeitouni A.G. (2015). Systemic inflammatory markers as independent prognosticators of head and neck squamous cell carcinoma. Head Neck.

[25] Pine J.K., Morris E., Hutchins G.G., West N.P., Jayne D.G., Quirke P. (2015). Systemic neutrophil-to-lymphocyte ratio in colorectal cancer: the relationship to patient survival, tumour biology and local lymphocytic response to tumour. Br J Cancer.

[26] Chang H., Gao J., Xu B.Q., Guo S.P., Lu R.B., Li G. (2013). Haemoglobin, neutrophil to lymphocyte ratio and platelet count improve prognosis prediction of the TNM staging system in nasopharyngeal carcinoma: development and validation in 3237 patients from a single institution. Clin Oncol (R Coll Radiol).

[27] Chan K.C. (2014). Plasma Epstein–Barr virus DNA as a biomarker for nasopharyngeal carcinoma. Chin J Cancer.

[28] Lo Y.M., Chan L.Y., Chan A.T., Leung S.F., Lo K.W., Zhang J. (1999). Quantitative and temporal correlation between circulating cell-free Epstein–Barr virus DNA and tumor recurrence in nasopharyngeal carcinoma. Cancer Res.

[29] Leung S.F., Chan K.C., Ma B.B., Hui E.P., Mo F., Chow K.C. (2014). Plasma Epstein–Barr viral DNA load at midpoint of radiotherapy course predicts outcome in advanced-stage nasopharyngeal carcinoma. Ann Oncol.

[30] Tang L.Q., Li C.F., Chen Q.Y., Zhang L., Lai X.P., He Y. (2015). High-sensitivity C-reactive protein complements plasma Epstein–Barr virus deoxyribonucleic acid prognostication in nasopharyngeal carcinoma: a large-scale retrospective and prospective cohort study. Int J Radiat Oncol Biol Phys.

